# Measuring the Impact of Research: Lessons from the UK’s Research Excellence Framework 2014

**DOI:** 10.1371/journal.pone.0156978

**Published:** 2016-06-08

**Authors:** Gobinda Chowdhury, Kushwanth Koya, Pete Philipson

**Affiliations:** Department of Mathematics and Information Sciences, Faculty of Engineering and Environment, Pandon Building, Camden Street, Northumbria University, Newcastle City Campus, Newcastle-upon-Tyne, NE2 1XE, United Kingdom; Universidad de Las Palmas de Gran Canaria, SPAIN

## Abstract

Impactful academic research plays a stellar role in society, pressing to ask the question of how one measures the impact created by different areas of academic research. Measuring the societal, cultural, economic and scientific impact of research is currently the priority of the National Science Foundation, European Commission and several research funding agencies. The recently concluded United Kingdom’s national research quality exercise, the Research Excellence Framework (REF) 2014, which piloted impact assessment as part of the overall evaluation offers a lens to view how impact of research in different disciplines can be measured. Overall research quality was assessed through quality of outputs, ‘impact’ and research environment. We performed two studies using the REF 2014 as a case study. The first study on 363 Impact Case Studies (ICSs) submitted in 5 research areas (UoAs) reveals that, in general, the impact scores were constructed upon a combination of factors i.e. quantity of quartile-one (Q1) publications, quantity and value of grants/income, number of researchers stated in the ICSs, spin-offs created, discoveries/patents and presentation of esteem data, informing researchers/ academics of the factors to consider in order to achieve a better impact score in research impact assessments. However, there were differences among disciplines in terms of the role played by the factors in achieving their overall scores for the ICSs. The outcome of this study is thus a set of impact indicators, and their relationship with the overall score of impact of research in different disciplines as determined in REF2014, which would in the first instance provide some answers to impact measures that would be useful for researchers in different disciplines. The second study extracts the general themes of impact reported by universities by performing a word frequency analysis in all the ICSs submitted in the five chosen research areas, which were substantially varied owing to their fields.

## Introduction

### The challenge of research evaluation

The National Science Foundation, European Commission which administers Horizon 2020, the UK government’s funding bodies like EPSRC, ESRC etc., and several research funding agencies have called for measuring the impact of research which they support [1–4]However, presently, no generalised impact evaluation frameworks exist. Research impact evaluation remains a major challenge despite the massive investment in research [5–7]. Database tools such as Science Citation Index, Web of Science, Scopus, Scholar, InCites, SciVal, h-index and Altmetrics make an effort to construct the quality of research through publication profile and citation profile or both [8–11]. However, these measures often remain problematic as a result of inadequate interpretations produced by mere numbers based on citation counts or access and/or download figures [12–15]. Alternative approaches such as web-impact metrics, societal impact and the multi-dimensional Leiden Manifesto have been proposed to alleviate some of these problems [9, 11, 16, 17]. Additionally there have been studies investigating the measure of research impact independent of time and discipline, and the practical implications of research [18, 19]. Nevertheless, we still do not have an agreed method or measures for assessing the impact of research.

The recently concluded REF2014 exercise introduced impact as a measure to assess quality of research across UK Universities in 36 disciplines over the period of 2008–2013. A similar exercise called the Research Assessment Exercise conducted in 2008 did not have impact in its assessment criteria. REF2014 measured the impact of research for each submitting university, under each of the 36 disciplines (called Units of Assessment, or UoA), and used the impact scores to distribute a significant amount of money to fund future research. Hence, the research reported in this paper aimed to understand the possible indicators for impact measures, and their influence or contributions to the overall score for research impact in REF2014. The outcome of this study is thus a set of impact indicators, and their relationship with the overall score of impact of research in different disciplines as determined in REF2014, which would in the first instance provide some answers to impact measures that would be useful for researchers in different disciplines. Furthermore, the findings could be useful for university management for future research assessment and evaluation exercises.

### What is the REF?

The REF 2014 was administered by a conjugated team of the Higher Education Funding Councils of England (HEFCE) and Wales (HEFCW), the Scottish Funding Council (SFC) and the Department for Employment and Learning (DEL), Northern Ireland [20]. It is conducted once every six or so years primarily aiming to evaluate the quality of research at HEIs, which further informs higher education funding bodies on assessing funding allocation for each higher education institution (HEI). Thus REF is an essential informant which the government uses to fund research and a barometer for the HEIs to measure their performance [20]. The REF, like its predecessor, the Research Assessment Exercise (RAE), also plays a significant role in an HEI’s league table scores, capability in securing funding from other sources (which amount to millions of pounds), national and international reputation, and thereby attracting students and high quality staff [21]. To give a general perspective, the current REF results are used to disburse 1.6 billion pounds of research money every year to UK higher education and research institutions until the next REF. The current results indicate some HEIs gained while others lost in REF2014 compared to their previous performance in the RAE2008. In one case, an HEI lost about 17.1% (£14.2 million) and in exceptional cases lost about 45%. One HEI gained 12.4% (£7.1 million) when compared to their RAE2008 scores [22–23]. As one would imagine, such drastic alterations can have serious consequences on the future of academic research in UK HEIs.

The REF process involves the HEIs choosing areas of research (called Units of Assessment or UoA), of the available 36 UoAs, which they wish to be evaluated upon and making a submission in a prescribed format—for each chosen UoA (see S1 Appendix Tables 1 & 2). In REF2014 the submissions were assessed by 1052 experts, of which 77% were academic and 23% users (individuals who make use of university research and collaborators outside academia in private, public or charitable sectors etc), under 36 expert sub-panel chairs working under the guidance of four main panel chairs to classify the quality of research as 4* (world leading), 3* (internationally excellent), 2* (recognised internationally), 1* (recognised nationally) and unclassified. The overall quality of research was assessed by the REF through quality of outputs (65% weightage) in terms of rigour, significance and originality with reference to international research quality; ‘impact’ (20% weightage), a newly introduced factor in REF2014 evaluating the ‘reach and significance’ of research on economy, society, culture, public policy or services, health, the environment or quality of life, beyond academia.; and research environment (15% weightage), in terms of ‘vitality and sustainability’ i.e. PhD completions, laboratory facilities and wider disciplinary contributions.

**Table 1:** Units of Assessment and their submission profile.

Unit of Assessment (UoA code)	External research income in ££	No. of researchers stated	No. of HEIs involved	No. of ICSs submitted
Clinical Medicine 1	6 billion	3926	31	383
Physics 9	2.4 billion	1773	41	203
General Engineering 15	1.26 billion	2553	62	291
CCMSLIM 36	64 million	1019	67	160
ADS 24	129.09 million	603	25	80
**Total**	**9.84 billion**	**9874**	**226**	**1117**

**Table 2:** Units of Assessment and potential range of impacts (REF, 2015).

Unit of Assessment (UoA code)	Range of impacts as described in Panel overview reports
Clinical Medicine 1	“included **increased life expectancy, reduced morbidity and improved quality of life** (for example, as a result of new drugs, vaccines, procedures, interventions and educational programmes**); reduced risk of future illness; improved knowledge transfer; improved efficiency and productivity of services; improved safety; improvement in the environment;** and a **significant contribution to industry, the UK economy and culture**. Many research programmes described in the impact case studies had led to a change in clinical guidelines and/or national policy, particularly via the UK National Institute for Health and Care Excellence (NICE).”
Physics 9/General Engineering 15	“impact received, including **impacts on the economy, public policy and services, society, culture and creativity, health, security, products, practitioners and professional services, and the environment**. Across all sub-panels a number of case studies were submitted based on **public engagement activity**. The sub-panels were impressed by the high degree of reach and significance of many of the examples of impact submitted.”
CCMSLIM 36	Impact was observed across **various aspects of civil society, cultural life, economic prosperity, education, policy making, public discourse and public services**.
ADS 24	“influencing professional practice in areas as diverse as **building design**, **the pedagogy of primary school teachers**, and the **training of elite athletes**. It is **influencing a wide range of public polices nationally and internationally in sustainable development**, **regulatory reform, poverty alleviation, child protection** and many more areas. It is doing so by **changing the climate of public opinion** as well as **directly influencing policy makers**. In some excellent examples the **status quo has been successfully challenged** and thereby the position of hitherto excluded or disadvantaged groups has been improved.”

Emboldened text indicates the elements theorised to have influenced impact scores

This study fundamentally focusses on the impact aspect of the REF evaluation. HEFCE broadly defines impact as “*an effect on*, *change or benefit to the economy*, *society*, *culture*, *public policy or services*, *health*, *the environment or quality of life*, *beyond academia*”. Impact was measured by expert panels on the basis of the impact case studies (ICSs) the HEIs had submitted (Examples), comparable to the Australian EIA (Excellence in Innovation for Australia) [24]. HEFCE provided guidance for HEIs advising a minimum number of ICS submissions based on the number of staff. Two ICSs were to be submitted per 14.99 of full time staff and an additional ICS for a further 10 and so on. The ICSs attempt to capture information on the context of research, its impact on multiple societal aspects (as defined above by HEFCE) during the 2008–2013 period, future strategic planning for support and outputs supporting the research that was undertaken, in addition to supporting statements as per REF’s guidelines. The REF panels assessed the narratives within the ICSs, mainly evaluating the ‘reach and significance’ of a particular research on multiple societal aspects [25]. Each ICS was consensually scored by three sub-panel members, out of which at least one member is a user. However, the impact aspect was subjected to much debate for its unclear definition (as defined above by HEFCE) and ambiguity right from the outset of REF 2014 [26]. The fundamental argument against ‘impact’ was that different disciplines have different impact criteria, which prevents a standard measure of impact and curtailment of academic freedom caused by external motivations when looking for impact outside academia [27–30]. However, HEFCE has indicated from the beginning that by virtue of different disciplines being categorised into panels it ensures fair evaluation of research, and within academic circles, the process was seen as strong peer-review and transparent [17].

The absence of a clear set of impact criteria created a significant degree of uncertainty amongst the submitting HEIs [30–31]. Rand Europe estimates that HEIs spent £55 million trying to effectively communicate their research’s impact for the REF2014, giving an impression of how important it is to the HEIs to score well and some even going to the extent of hiring specialist writers or consultants [32], which was criticised by the REF saying “the lack of academic language and emphasis hindered the ability of the sub-panel to judge against criteria” [26, 33].

### REF’s impact evaluation challenges

A recent report by the RAND Corporation (2015) commissioned by HEFCE, after the publication of REF 2014 results, reveals several challenges faced by impact assessors during the evaluation of the ICSs and solicits HEFCE to provide clear guidelines to both the submitting institutions and evaluators [34]. The report indicates that 34% and 30% of the impact assessors felt the evaluation process was unreliable in assessing the criterion of significance and reach respectively. Several challenges were faced by the evaluators, such as: panellists finding it difficult to connect between impact and underpinning research, assessing ICSs which fell on the funding threshold between 2-star and 3-star, restricted access to evidence corroborating impact and general scoring discord.

*“In areas of Main Panels B and D in particular it was noted that whilst some panellists found it easy to assess impact when it was clearly 4 star or 1 star*, *they found it harder to assess the middle bands (2 and 3-star)*, *especially in regards to reach and significance*. *It was thought that the small sample which was calibrated included high scoring case studies and low scoring case studies but did not allow panel members to ‘examine some of the more nuances around…what may be a very good 3-star impact case study’*. *(REF 2015 for Main Panel A)*.*”* [34]

In the evaluation, some panels used half stars, thereby using 8 as opposed to four grades, in order to address the challenges where an ICS falls between two star grades, i.e. between 2 and 3, and 3 and 4, etc. This also created some problems. For example, as the RAND Corporation report (2015) suggests because of the virtual 8-star approach used to evaluate the ICSs from a granular level, in one panel all the ICSs which had scored between 4-star and 8-star were all awarded 4-star [34].

*“…*..*all main panels used 1/2 stars to create more granularity*. *In addition*, *areas of Main Panel A developed a framework of up to 8 stars when structuring their discussions about scores*. *The rationale behind this was that there were different levels within each star*, *and it ensured that 4-star impacts were not downgraded due to comparisons with exceptional examples (a problem discussed below in Section 1*.*2*.*3)*. *In Main Panel A*, *when awarding the scores*, *case studies scoring 4–8 were all graded as 4-star*.*”* [34]

Therefore, one may theorise that in this panel many ICS that were graded 4* would have been below 4* if only 4 as opposed to an 8 star scale was used. In its entirety, the evaluation of impact in our understanding remains highly subjective with its challenges mainly grouped into three main clusters; volume and complexity of the REF, difficulties in consensual scoring and the potential inflation of scores.

### Objective

As the indicators on which the ICSs were measured have not been mentioned clearly either before or after the exercise, our aim was:

1. To understand which set of factors the HEIs may have used to describe their ICSs in different disciplines, and thereby identify a set of possible impact indicators;
2. To understand what influence these identified factors had on the final score achieved by the HEIs on their research impact.

Clarity on these two issues would assist researchers and HEIs to understand how their research was assessed for the purpose of making an improved submission for assessment in the future and hence, increase the chances of acquiring funding.

Impact scores were used for the disbursal of £320 million out of the £1.6 billion in research funds each year until the next REF by the UK government. As the government disburses funds based on these scores, a clear understanding of the impact measures will help HEIs secure funding through which better research capabilities can be created, therefore shaping the future of research in different disciplines, and thus the entire research and scholarship activities in the country. This would also provide a general understanding of the various factors contributing to the impact measures of research in different disciplines, rather through publications only.

### The Chosen Disciplines

As stated in section 3.1, this study evaluates a selected set of ICSs submitted under five randomly chosen UoAs, namely Clinical Medicine, Physics, General Engineering, Communication, Cultural and Media Studies and Library and Information Management (CCMSLIM), Anthropology and Development Studies (ADS). A total of 226 HEIs submitted ICSs in these five UoAs and they reported a total amount of £9.84 billion research spending received through several funding sources, involving 9874 researchers in the REF period (2008–2013) (Table 1).

A total of 1117 ICSs submitted by the HEIs were subsequently evaluated by the REF2014 panels against the ‘reach and significance of research’ and categorised them into the quality standards in terms of percentage in: 4*, 3*, 2*, 1* and unclassified. However, it still remains unclear how each ICS was evaluated, other than a range of impact factors identified from panel executive reports of the selected UoAs in Table 2, which could be classified as the qualitative factors indicating research impact.

With quality related research funding, research quality and knowledge at stake, it becomes essential to understand the factors which constructed the impact scores to better assist researchers to improve their submission for the next REF in 2021, and subsequently start a dialogue with HEFCE to mutually agree on deliverables which measure the true quality of research [35].

## Methods

### Study Design

The study analysed 363 ICSs submitted by the top 5 and bottom 5 performers in four UoAs namely, Clinical Medicine, Physics, General Engineering,; and top 10 and bottom 10 performers in two UoAs, namely, CCMSLIM and ADS. The UoAs were chosen randomly from different panels to understand disciplinary differences in impact. The sample sizes (top 5/10 and bottom 5/10) were chosen to understand the anatomy of the submitted impact case studies and why were they ranked in the REF as top 5/10 and bottom 5/10. Clinical medicine belonged to Panel A, Physics and General Engineering were from Panel B, ADS from Panel C and CCMSLIM from Panel D. The number of ICSs chosen for each UoA is as follows: 92 in clinical medicine; 72 ICSs in physics; 89 ICSs in general engineering; 63 ICSs in ADS; and 47 in CCMSLIM. The chosen impact case studies in each UoA comprised of all the ICSs’ submitted by the top 5 and bottom 5 HEIs, except for ADS and CCMSLIM, in whose cases it was top 10 and bottom 10, based on their impact profile scores of 4*. Top 10 and bottom 10 were chosen in ADS and CCMSLIM as the number of ICS were relatively less. To clarify, an HEI with impact score of 100 has had all its ICSs rated 4*, one with score of 90 has had 90% of its ICSs rated 4*, and so on. Hence, determining the score of each ICS was not possible, nullifying bias. It is also important to note that, according to the REF guidelines, the number of case studies required for submission is determined by the number of full-time staff returned for the UoA (REF, 2014). However, this did not affect the analysis as each ICS was individually examined by the investigators. As there was no set standard for reference, except for ‘reach and significance’ to examine the ICSs for quality, it was impossible to rely on a specific factor. However, manually reviewing all the case studies from the chosen UoAs to understand their content revealed a number of factors which were used as parameters for this study:

1. No. of Q1 publications–According to REF guidelines, every ICS must refer to outputs (maximum 6) where the research has been published. We considered how many of these were in Q1 journals in relevant fields. A Q1 journal possesses a high impact factor and number of citations in a specific subject area. Metrics in general create narrow interpretations of research quality, as they are solely focussed on number of citations of a paper in a particular journal [16, 17]. Despite this fact, our curiosity drove us to examine any metric differences of the papers refereed in the ICSs, which would indicate an author’s preference of a journal to publish their research and an ICS’s impact score prospects.
2. No. of researchers stated–The number of researchers stated in each ICS was considered as a potential determinant as quantity of researchers involved in a project indicates the amount or volume of funding, collaboration and support.
3. Total income stated in the ICS–The income stated in each ICS earned through grants, patents, spin-offs, product sales and investments etc. indicating the economic impact of the research, in addition to demonstrating that the research has been peer-reviewed.
4. External research income stated for the REF–The income stated by the HEI in each UoA for the REF submission demonstrates a research’s support in the academic community as a result of peer-review. This is different from (c) as the REF requires the HEIs to explicitly mention external grant and in-kind income only.
5. Number of grants/patents/discoveries/spin-offs stated in each ICS–The quantity of grants received, patents acquired, discoveries made and spin-offs found as stated in the ICS.
6. How were the grants/income/patents/discoveries/spin-offs presented?–Manual examination of the case studies revealed a contrasting structural difference in how HEIs presented their esteem factors in ICSs. In addition to having stated none, a number of ICSs explicitly stated their achievements with bold headings. However, for many, we had to search for the information using terms such as ‘patents’, ‘discoveries’, ‘fund’, ‘grant’, ‘support’, ‘spin-offs’ and ‘sponsor’. Data was ranked as ‘explicitly stated’, ‘searched’ and ‘not stated’.

### Data

The ICSs and Excel spreadsheets consisting of HEIs performance indicators were downloaded from the publicly available REF’s results website (REF, 2014). Thomson Reuter’s Journal Citation Reports^®^ service assisted in establishing a journal’s impact factor [36]. The ICSs were grouped according to their HEIs impact scores and the variables considered were coded into IBM SPSS Statistics 22, transferred as comma-separated value (csv) files to R to investigate the following rationale:

– What’s the role of referenced Q1 journal articles in ICSs on ICS scores?
– What are the implications of income on ICS scores?
– Is there a relationship between the number of researchers mentioned in the ICSs and the ICS scores?
– Do patents/discoveries impact the ICS scores?
– Do spin-offs impact the ICS score?
– Did different types of presentation of esteem factors such as patents/discoveries/income affect the ICS scores?
– Do more grants result in higher ICS scores?

### Statistical Analysis

The chosen variables from the 363 ICSs were considered as independent variables and the impact scores, which are restricted values between 0 and 100, were considered as the dependent variable. Additionally, impact scores were also scaled down by dividing the impact score by 100, due to the presence of a number of ICSs scored as 0 and 100. A beta-regression method coupled with a backward elimination process using the betareg package in R was applied to analyse the effect of multiple variables on the impact scores [37–40]. The removal of each variable with the highest p-value at each step of the model verification process was used as the criteria for backward elimination, until all variables with a significant p-value remain in the model. A units based transformation was applied to the income based variables as their values were large. For example, an HEI which had stated its income as £145,697,136 was transformed to 1.45697136 as high values tend to affect the analysis. However, a comparison of the transformed model and the untransformed model revealed no difference, assuring stability.

The exponential coefficients as determined by the beta-regression analysis correspond to the log-odds. For example, in a hypothetical case, if the coefficient of Q1 publications is 0.1, then we take exp(0.1) = 1.1, which suggests, for each additional Q1 publication, the odds of having a higher impact score increases by 11%.

### Qualitative analysis

All the ICSs submitted by HEIs in the chosen five UoAs were downloaded from the REF’s website and were analysed by a text query function to extract the top 100 themes using Nvivo 10. The text query function was set to a minimum word length of three letters and was synonymously grouped to include words which fall into the same definition bracket. A manual filtration process was applied to remove commonly occurring sentence fillers. A word cloud was built on the basis that a word with more references in the text would appear and a word with fewer references in the text would appear smaller.

## Results

It appears that there was a difference in variables in ICSs belonging to high scoring HEIs and low scoring HEIs. However, the external research income stated by the HEIs for the REF appears to be a consistent and strong determinant of the ICS scores.

### Clinical medicine

The number of Q1 publications appeared to be the most consistent variable in determining the ICS scores in clinical medicine as in Table 3. The odds of having a higher impact score increases by 12.6% for every additional Q1 publication stated in the ICSs.

**Table 3:** Coefficients of variables in the beta-regression model of clinical medicine.

Variable	Estimate	Standard error	p-value
Q1 pubs	0.238	0.076	0.001

#### Themes in Clinical Medicine

**Fig 1:**
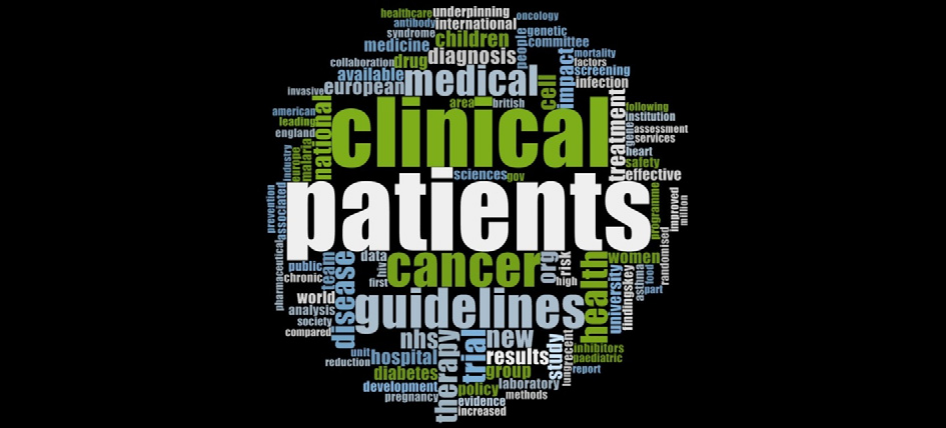
Impact themes in Clinical Medicine.

Fig 1 showcases the various themes of impact extracted through a word frequency analysis from all the ICSs submitted by HEIs under the clinical medicine UoA to the REF 2014. Research into the wellbeing of patients, paediatric medicine, oncology, genetics, diabetes, heart health and public health etc appear as the fundamental themes of research across UK HEIs in clinical medicine.

### Physics

The quantity of researchers, research income stated for the REF and spin-offs created appeared to be the most consistent variables in determining the ICS scores in physics as in Table 4. The odds of having a higher impact score increases by 11%, 18% and 16% for every additional researcher, income and spin-off, respectively, stated in the ICSs.

**Table 4:** Coefficients of variables in the beta-regression model of physics.

Variable	Estimate	Standard error	p-value
No. of researchers	0.094	0.038	<0.01
Income stated for REF	0.592	0.171	<0.01
Spin-offs	0.485	0.154	<0.01

#### Themes in Physics

**Fig 2:**
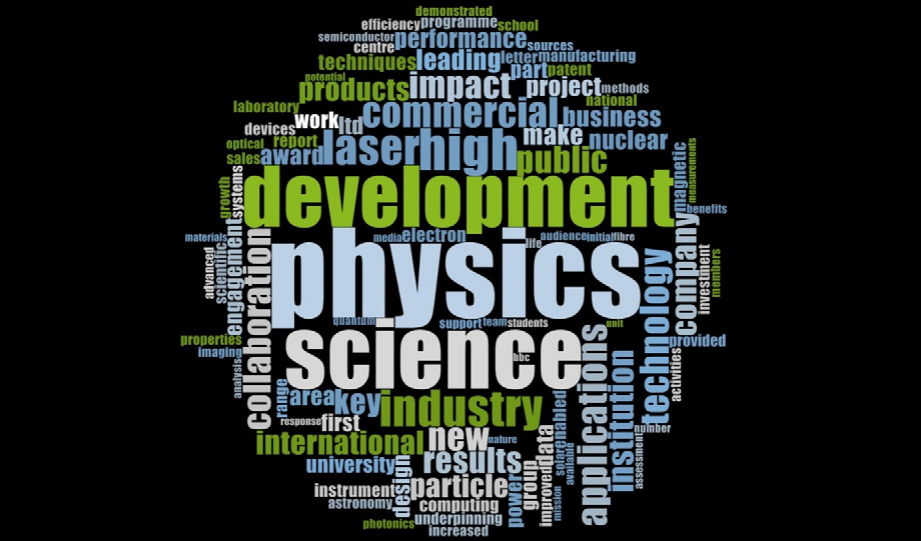
Impact themes in Physics.

Fig 2 showcases the various themes of impact extracted through a word frequency analysis from all the ICSs submitted by HEIs under the physics UoA to the REF 2014. Research into high energy and nuclear physics, astronomy, solar studies, material sciences and research yielding high commercial potential etc appear as the fundamental themes of research across UK HEIs in physic.

### General Engineering

The number of Q1 publications and research income stated for the REF appeared to be the most consistent variables in determining the ICS scores in general engineering as in Table 5. The odds of having a higher impact score increases by 13% and 45% for every additional Q1 publication and income, respectively, stated in the ICSs.

**Table 5:** Coefficients of variables in the beta-regression model of general engineering.

Variable	Estimate	Standard error	p-value
Q1 pubs	0.259	0.073	<0.01
Income stated for REF	1.516	0.221	<0.01

#### Themes in general engineering

**Fig 3:**
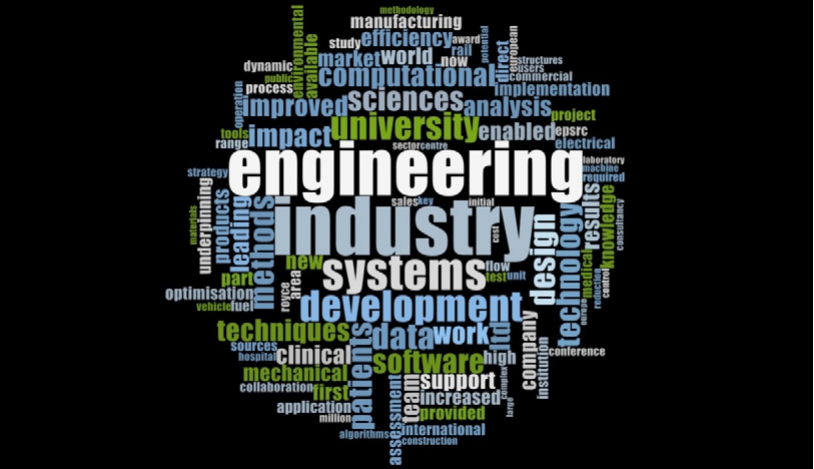
Impact themes in General Engineering.

Fig 3 showcases the various themes of impact extracted through a word frequency analysis from all the ICSs submitted by HEIs under the general engineering UoA to the REF 2014. Research yielding high commercial potential, building of software, improving efficiency of machines, sustainability and clinical procedures etc appear as the fundamental themes of research across UK HEIs in general engineering.

### ADS

The research income stated for the REF appeared to be the most consistent variables in determining the ICS scores in ADS as in Table 6. The odds of having a higher impact score increases by 13.5% for additional income stated in the ICSs

**Table 6:** Coefficients of variables in the beta-regression model of ADS.

Variable	Estimate	Standard error	p-value
Income stated for REF	0.303	0.075	<0.01

#### Themes in ADS

**Fig 4:**
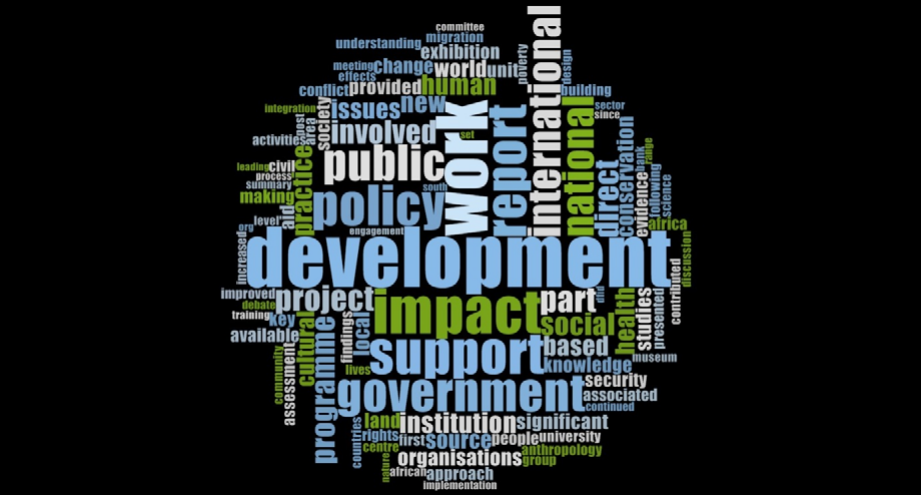
Impact themes in ADS.

Fig 4 showcases the various themes of impact extracted through a word frequency analysis from all the ICSs submitted by HEIs under the anthropology and development studies UoA to the REF 2014. Research into improving human conditions, increasing the efficiency of government funded entities, migration and conservation etc appear as the fundamental themes of research across UK HEIs in ADS.

### CCMSLIM

The total income mentioned in the ICS, research income stated for the REF and quantity of grants appeared to be the most consistent variables in determining the ICS scores in CCMSLIM as in Table 7. The odds of having a higher impact score increases by 16%, 13% and 12% for additional income mentioned in the ICS, income mentioned for REF and grants respectively.

**Table 7:** Coefficients of variables in the beta-regression model of CCMSLIM.

Variable	Estimate	Standard error	p-value
Income mentioned in ICS	0.485	0.249	0.05
Income stated for REF	0.303	0.075	<0.01
No of grants	0.173	0.073	0.01

#### Themes in CCMSLIM

**Fig 5:**
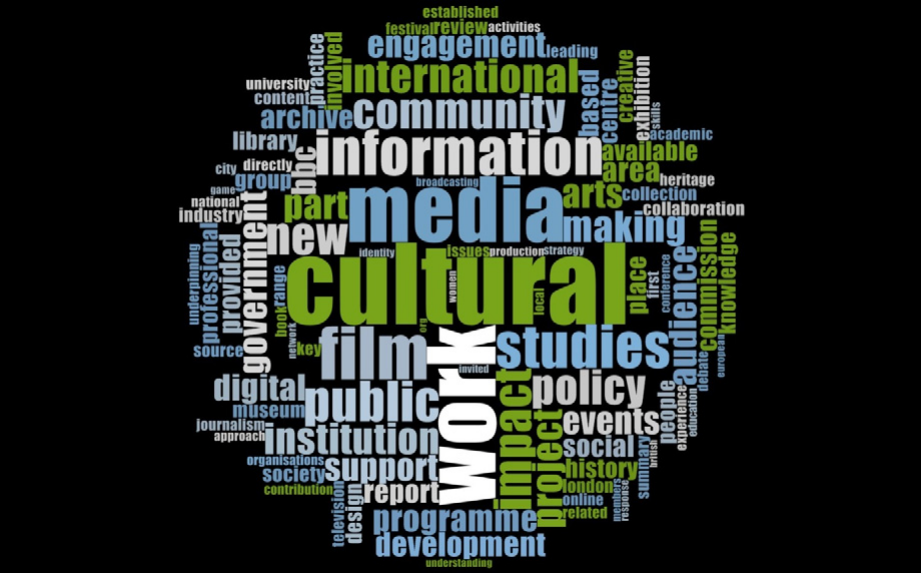
Impact themes in CCMSLIM.

Fig 5 showcases the various themes of impact extracted through a word frequency analysis from all the ICSs submitted by HEIs under the communication, cultural and media studies, library and information management UoA to the REF 2014. Research into culture, media studies, libraries, information retrieval, collection building, films, policy studies, heritage conservation and community studies etc appear as the fundamental themes of research across UK HEIs in CCMSLIM.

## Discussions

### Informing researchers/academics and HEI administration

The findings indicate the different variables researchers/academics, especially UK researchers/academics in the examined fields have to consider in order to secure a good impact score in addition to the qualitative indicators mentioned by the REF panels for specific disciplines mentioned by the REF (Table 8, Figs 1–5).

**Table 8:** Various variables affecting ICS scores in different disciplines.

UoA→Factors↓	Clinical medicine	Physics	General Engg.	CCMSLIM	ADS
Q1 pubs	*		*		
No. of researchers		*			
Income presentation					
Esteem data presentation					
Income stated in ICS				*	
Income stated for REF		*	*	*	*
No. of grants				*	
No. of spin-offs		*			

* Variables affecting the impact scores in different disciplines.

Thus HEI administrators will be persuaded to submit ICSs which excel in the variables examined, in addition to the factors highlighted in Table 2. Generally, the findings have the potential to inform an HEI’s recruitment and research strategy.

The findings can also inform specific strategies for submission of ICSs in specific disciplines:

HEIs submitting to the Clinical Medicine UoA should demonstrate that their research improves the quality of life, life expectancy, reduces morbidity and risk of future illness, improves knowledge transfer, efficiency, productivity of services and safety, and significantly contributes to the industry and UK economy. Their impact case studies should also explicitly focus on research income and publications made in high impact journals.

HEIs submitting to the Physics and General Engineering UoAs should demonstrate public engagement activities, impacts on economy, society, services, culture, creativity, health, security, products, practitioners, professional services and the environment. Their impact case studies should also explicitly focus on the level of collaboration, research income secured, spin-offs created and publication in high impact journals.

HEIs submitting to the CCMSLIM UoA should demonstrate that their research has impact across civil society, cultural life, economic prosperity, education, policy making, public discourse and public services. Their impact case studies should also explicitly focus on income generated and grants secured.

HEIs submitting to the ADS UoA should demonstrate that their research influences sustainable development, regulatory reform, poverty alleviation, child protection, a wide range of public policies internationally and changing general public opinions. Their impact case studies should also research income secured.

### Income and size bias?

The external income stated by the HEIs for the REF appears to be a consistent and strong determinant of the ICS scores. Further investigations into the remaining 31 UoA are required to verify the inclination of better ICS scores towards high income and staff sizes.

### Presentation of data in ICS

Our study is limited to 363 ICSs in five units of assessment belonging to top and bottom five in all chosen UoAs, except for CCMSLIM, in which case it was top and bottom 10. However, every detail of the ICSs was manually examined in order to understand which factors may have affected its impact score, resulting in questions being raised regarding evaluation, definition of funding sources, income and procedures. The HEIs when stating their sources of income, had to state it through a code allocated by the REF. The definitions for various funding sources and their corresponding codes were ambiguous. For example, the Wellcome Trust and British Heart Foundation are UK based charities which support health research. How does one identify them between funding code 2 (UK based charities) and 14 (Income from specific bodies that fund health research)? In terms of income, does external investment or selling of a spin-off count as income and how is intra-university funding stated in the ICSs? As funding played a significant role in constructing the impact scores, it is important to clarify these questions.

Lack of clarity on these issues resulted in HEIs submitting their ICSs in different formats, which may have had implications during evaluation. This finding corroborates the REF panels’ executive summary which notes that lower impact profile HEIs had been inferior at presenting information explicitly and not according to prescribed formats [41], which was also observed in the RAND study [34]. This suggests that HEFCE needs to provide clear submission guidelines to assist HEIs in making proper submissions. A number of ICSs stated how their research significantly saved costs, had a human impact and drove policy internationally. Quantifying these factors may help HEIs submit better quality ICSs. A few disparities were also noted in the ICSs in addition to unstructured presentation of assets. Although the REF had instructed the HEIs to mention only the institution’s share of a certain fund, ICSs, especially in the lower ranks presented the whole pot. Quite a number of HEIs have stated the source of funding, but haven’t mentioned the value. As the findings indicate a relationship between funding and impact scores, it is deemed necessary to include the value of funding.

### Further qualitative and quantitative analysis of ICSs

This study mainly focussed on studying the quantifiable implicit factors in the ICSs. During manual examination of the ICSs, it was recognised that a qualitative analysis using advanced machine learning and linguistic analysis would provide significant insights into the qualitative features of the ICSs that are characteristic of each discipline. Additionally, a multivariate analysis of the variables using various visualisation techniques would provide a deeper understanding on the evaluation of ICSs.

## Conclusion

This study for the first time identifies various factors or indicators of research impact and their overall influence on the impact scores as evaluated in the REF 2014. Consummately, the findings indicate that the REF scores were constructed on a range of factors, from the variables considered by us in addition to the qualitative factors indicated by the REF panel reports (Table 2). The results will be useful for university administrators to choose ICSs excelling in the identified factors, encourage researchers and academics to produce high quality research that have social and economic implications. Additionally, this study appeals to the research evaluating agencies to provide appropriate guidelines based on a set of generic and discipline-specific qualitative and quantitative factors that would help HEIs make better ICS submissions for future evaluation exercises.

## Transparency and Data-Sets

The datasets are publicly available at http://nrl.northumbria.ac.uk/25671/. The authors designed and investigated the study using the REF’s data and Thomson Reuters’s Web of Science^®^ and Journal Citation Reports^®^ which are openly available in the public domain through their respective websites. The authors affirm that the manuscript is an honest, accurate, transparent account of the study being reported; that no important aspects of the study have been omitted; and that any discrepancies have been disclosed.

## Supporting Information

S1 Appendix Units of assessment and impact template. (DOCX)
