# Supplementary material for: Measuring the Impact of Research: Lessons from the UK’s Research Excellence Framework 2014
Source: PLoS One. 2016 Jun 8;11(6):e0156978. doi: 10.1371/journal.pone.0156978 (PMC4898824; doi:10.1371/journal.pone.0156978)
Supplement: S1 Appendix — (DOCX) [file pone.0156978.s001.docx]

**S1 Appendix. Units of assessment and impact template**

Table 1. Units of assessment

| **Main panel** | **Unit of assessment** | |
| --- | --- | --- |
| A | 1 | Clinical Medicine |
|  | 2 | Public Health, Health Services and Primary Care |
|  | 3 | Allied Health Professions, Dentistry, Nursing and Pharmacy |
|  | 4 | Psychology, Psychiatry and Neuroscience |
|  | 5 | Biological Sciences |
|  | 6 | Agriculture, Veterinary and Food Science |
| B | 7 | Earth Systems and Environmental Sciences |
|  | 8 | Chemistry |
|  | 9 | Physics |
|  | 10 | Mathematical Sciences |
|  | 11 | Computer Science and Informatics |
|  | 12 | Aeronautical, Mechanical, Chemical and Manufacturing Engineering |
|  | 13 | Electrical and Electronic Engineering, Metallurgy and Materials |
|  | 14 | Civil and Construction Engineering |
|  | 15 | General Engineering |
| C | 16 | Architecture, Built Environment and Planning |
|  | 17 | Geography, Environmental Studies and Archaeology |
|  | 18 | Economics and Econometrics |
|  | 19 | Business and Management Studies |
|  | 20 | Law |
|  | 21 | Politics and International Studies |
|  | 22 | Social Work and Social Policy |
|  | 23 | Sociology |
|  | 24 | Anthropology and Development Studies |
|  | 25 | Education |
|  | 26 | Sport and Exercise Sciences, Leisure and Tourism |
| D | 27 | Area Studies |
|  | 28 | Modern Languages and Linguistics |
|  | 29 | English Language and Literature |
|  | 30 | History |
|  | 31 | Classics |
|  | 32 | Philosophy |
|  | 33 | Theology and Religious Studies |
|  | 34 | Art and Design: History, Practice and Theory |
|  | 35 | Music, Drama, Dance and Performing Arts |
|  | 36 | Communication, Cultural and Media Studies, Library and Information Management |

Table 2. Impact Template

| **Institution:** |
| --- |
| **Unit of Assessment:** |
| **a. Context** |
| **b. Approach to impact** |
| **c. Strategy and plans** |
| **d. Relationship to case studies** |
